# Mislocalization of the exitatory amino-acid transporters (EAATs) in human astrocytoma and non-astrocytoma cancer cells: effect of the cell confluence

**DOI:** 10.1186/1423-0127-19-10

**Published:** 2012-02-01

**Authors:** Karine Varini, Amal Benzaria, Nadira Taïeb, Coralie Di Scala, Amanda Azmi, Soraya Graoudi, Marc Maresca

**Affiliations:** 1 Interactions cellulaires neuroimmunes et pathologies du système nerveux central, CRN2M, CNRS UMR 6231, University of Aix-Marseille 2 and Aix-Marseille 3, Faculté de Médecine - Secteur Nord, Université de la Méditerranée, Marseille, France; 2 Aix-Marseille Université, Faculté des Sciences de St-Jérôme, 13397 Marseille Cedex 20, France

**Keywords:** Astrocytoma, Cancer, GLAST, GLT-1, Glutamate, EAAT, Mislocalization, STTG-1

## Abstract

**Background:**

Astrocytomas are cancers of the brain in which high levels of extracellular glutamate plays a critical role in tumor growth and resistance to conventional treatments. This is due for part to a decrease in the activity of the glutamate transporters, i.e. the Excitatory Amino Acid Transporters or EAATs, in relation to their nuclear mislocalization in astrocytoma cells. Although non-astrocytoma cancers express EAATs, the localization of EAATs and the handling of L-glutamate in that case have not been investigated.

**Methods:**

We looked at the cellular localization and activity of EAATs in human astrocytoma and non-astrocytoma cancer cells by immunofluorescence, cell fractionation and L-glutamate transport studies.

**Results:**

We demonstrated that the nuclear mislocalization of EAATs was not restricted to astrocytoma and happened in all sub-confluent non-astrocytoma cancer cells we tested. In addition, we found that cell-cell contact caused the relocalization of EAATs from the nuclei to the plasma membrane in all human cancer cells tested, except astrocytoma.

**Conclusions:**

Taken together, our results demonstrated that the mislocalization of the EAATs and its associated altered handling of glutamate are not restricted to astrocytomas but were also found in human non-astrocytoma cancers. Importantly, we found that a cell contact-dependent signal caused the relocalization of EAATs at the plasma membrane at least in human non-astrocytoma cancer cells, resulting in the correction of the altered transport of glutamate in such cancer cells but not in astrocytoma.

## Background

Among adult brain tumors, gliomas are the most common form, accounting for more than 70% of the brain cancer [1,2]. Gliomas arise from the malignant transformation of glial cells, mainly astrocytes, oligodendrocytes and ependymal cells. Astrocytomas are the most frequent and malignant form of gliomas and are associated generally to a poor prognostic [1,2]. Thus, meta-analysis have showed that 1 year survival rates of patients affected by astrocytomas is around 40% and that conventional treatments (i.e. surgery, radiotherapy and chemotherapy) only slightly increase the survival (from 40 to 46%, respectively) [3].

In vivo and in vitro experiments have showed that the growth, invasion and resistance to treatment of astrocytomas are dependent of an altered handling of the glutamate by malignant astrocytes [4-7] although other factors, such as tissue hypoxia and modification of surface antigens, could participate [8-11].

Physiologically, normal astrocytes are responsible for the recapture of the glutamate released by glutaminergic neurons during the synaptic communication. This recapture is essential for the termination of the synaptic transmission and to prevent neuronal damage caused by high excitotoxic extracellular glutamate concentrations [12]. Glutamate uptake by astrocytes takes place mainly through two high affinity sodium-dependent excitatory amino-acid transporters (EAAT), i.e. EAAT1/GLAST and EAAT2/GLT-1, isoform's expression by normal astrocytes being specific of brain area [12]. Contrary to normal astrocytes that absorb more glutamate than they secrete it, malignant astrocytes are responsible for a high secretion of glutamate at the vicinity of the tumor [4,6,7]. This major difference in the handling of the glutamate by normal and malignant astrocytes is due to alterations of the activity/expression of glutamate transporters, i.e. excitatory amino-acid transporters (EAATs) and the cystine-glutamate exchanger (X_c_^-^). EAATs are responsible for the absorption of glutamate whereas X_c_^- ^is involved in the secretion of glutamate and the entry of L-cystine, a precursor of glutathione. In normal astrocytes the activity of EAATs is higher than the activity of X_c_^-^, resulting in a net absorption of glutamate. Conversely, malignant astrocytes display a defect in the EAAT-dependent absorption of glutamate and an increase in X_c_^-^-dependent secretion of glutamate, causing the net secretion of the excitatory amino-acid observed in astrocytomas. Previous study elegantly showed that the defect of EAATs activity in human astrocytomas and all human astrocytoma cell lines (including STTG-1 cells) is due to the mislocalization of the transporters into the nuclei [13]. Thus, EAATs were found in the nuclei of all human astrocytoma cell lines tested and in astrocytoma biopsies, making of STTG-1 a good in vitro model to study EAATs mislocalisation in astrocytoma.

The resulting high extracellular concentration of glutamate at the vicinity of the tumor has major implication both in terms of pathophysiology and cancer biology [4,6,7]. Thus, the glutamate secreted by astrocytomas induces the death of normal brain cells surrounding the tumor through activation of the ionotropic glutamate receptor (NMDA) and excito-toxicity, making more space for the tumor to expend. Secreted glutamate is also responsible for epilepsy and other neurologic disorders associated with astrocytomas. Moreover, the secretion of glutamate by malignant astrocytes allows the entry of L-cystine through the X_c_^- ^exchanger, leading to an increase in the intracellular concentration of glutathione and to an increase in the resistance of astrocytomas to oxidative stress caused by radiation or chemo-therapy. Finally, the secreted glutamate stimulates the division of malignant astrocytes by activating metabotropic glutamate transporters through para- and autocrine action [14].

Based on the high dependency of astrocytomas to extracellular glutamate, new treatment strategies have been developed to strike the tumors at the level of the glutamate transporters and receptors. Thus, inhibitors of X_c_^- ^exchanger have been shown to decrease the growth, invasion and the resistance of astrocytomas to radiation and chemo-therapy by limiting the intracellular concentration of glutathione [15]. Antagonists of metabotropic glutamate receptors have been also used successfully to limit the growth of astrocytomas by blocking the para-/autocrine stimulation of the growth of tumor cells by secreted glutamate [16,17]. However, to date, no strategies have been developed to correct the defect of EAATs-mediated absorption of glutamate observed in human astrocytomas, the cellular or molecular event responsible for the nuclear localization of EAATs being still uncharacterised. Correcting the mislocalization of EAATs will theoretically reverse the net secretion of glutamate by malignant astrocytes into a net absorption and thus will deprive the tumor of the extracellular glutamate essential for its growth, invasion and resistance.

Although it has been demonstrated that cancer cells of non-central nervous system origin also express EAATs and could secrete glutamate through X_c_^- ^activity [18,19], the activity/localization of EAATs in non-astrocytoma cancers were not evaluated. In the present study, we looked at the cellular localization and activity of EAATs in human cancer cells originating from various tissues and found that the nuclear mislocalization of EAAT and the associated altered glutamate handling are not limited to astocytoma cancer and happens in many human cancer cells. We also found that the mislocalization of EAATs could be corrected by cell contact in all human cancer cells tested, except astrocytoma.

## Methods

### Ethical treatment of animals

All work involving animal have been conducted in accordance with the European Communities Council Directive of 24 November 1986 (86/609/EEC) and with the local committee's recommendations (C-13-055-6, Aix-Marseille University).

### Human cancer cells and primary cell cultures

Human cells used in this study are summarized in Table 1. All cells were from ATCC, except SH-SY5Y and STTG-1 cells which were obtained from ECACC and HOG cells which were initially isolated from a surgically removed human oligodendroglioma [20] and that were kindly provided by Drs. López-Guerrero and Bello-Morales (CSIC-UAM, Cantoblanco, Madrid, Spain). All human cells were routinely cultured on 75 cm^2 ^flasks in Dulbecco's modified essential medium (DMEM) supplemented with 2 mM glutamine, 10% (v:v) fetal calf serum (FCS) and 100 μg/mL streptomycin and 100 U/mL penicillin. Flasks were maintained in a humidified incubator at 37°C with 95% air and 5% CO_2_. Primary cultures of cortical astrocytes were prepared from new born rats as previously described [21,22].

**Table 1:** List of human cancer cells used in this study.

Name	ATCC number	Cancer type	Organ/cell type
AGS	CRL-1739	Adenocarcinoma	Stomach/Epithelial cell

Caco-2	HTB-37	Adenocarcinoma	Colon/Epithelial cell

HeLa	CCL-2	Adenocarcinoma	Cervix/Epithelial cell

HOG	-	Oligodendrocytoma	Brain/Oligodendrocyte

HT-29	HTB-38	Adenocarcinoma	Colon/Epithelial cell

SH-SY5Y	CRL-2266	Neuroblastoma	Brain/Neuron

STTG1	CRL-1718	Astrocytoma	Brain/Astrocyte

### Immunofluorescence microscopy and image analysis

Cells were seeded onto glass coverslips at an initial cell density of 100,000 cells per cm^2^. Sub-confluent (typically 1-2 days after seeding) and confluent cells (typically 7-10 days post-seeding) were washed once time in phosphate buffer saline (PBS) and were fixed for 10 min at room temperature with 4% paraformaldehyde diluted in PBS. Coverslips were then washed three times in PBS and incubated for 1 h at room temperature in saturation/permeabilisation buffer containing 2% (w:v) bovine serum albumin and 0.1% Triton X-100 diluted in PBS. After three washes with PBS, primary antibodies directed against EAAT1/GLAST or EAAT2/GLT-1 (diluted in PBS according to manufacturers) were added for 1 h at room temperature. Primary antibodies used in this study were goat anti-EAAT1 (AB1782) and anti-EAAT2 (AB1783), obtained from Chemicon, directed against amino-acids from the C-terminal part of EAAT1 or 2, and rabbit anti-EAAT1 (sc-15316) and anti-EAAT2 (sc-15317) obtained from Santa Cruz and directed against amino-acids from the N-terminal part of EAAT1 or 2. Cells were also stained with primary antibodies (all from Santa Cruz) directed other antigens specific of particular cell compartments according to manufacturer instructions. After three washes with PBS, primary antibodies were detected using specific secondary antibodies conjugated to Alexa-488 (Invitrogen) (1:200 dilution in PBS) for 1 h at room temperature. In some cases, actin was stained using phalloidin-TRITC (Sigma, diluted 1:2000). Finally, coverslips were washed six times with PBS and were mounted in Vectashield medium containing the nuclear stain DAPI, sealed with nail varnish and viewed using a fluorescence microscope (Leica). Fluorescence intensity determination and image analysis were performed using Image J (NIH) and measured as arbitrary fluorescence values based on the mean numbers of pixels for each channel. After background subtraction, total fluorescence from individual cells and from nuclei were determined as a region of interest (ROI) using Image J software. The nuclear signal was directly measured by making an ROI around the nuclei whereas total cell fluorescence was measured by making an ROI around the whole cells. At least 100 different cells were analysed per conditions. Negative control slides not incubated with primary antibodies were used to set base parameters for each series of slides, which was maintained during visualisation, ensuring the detected signal was specific.

### Cell fractionation and western-blot analysis

Cellular localization of EAATs was studied using detergent-based cell fractionation kit from Pierce. Cells were seeded onto 10 cm^2 ^plastic Petri dishes and cell fractionation was done according to manufacturer. Briefly, attached cells were washed three time with ice-cold PBS^++ ^and then scraped in ice-cold PBS. Cells were transferred in microcentrifuge tubes and pelleted by centrifugation at 500 × *g *for 5 min. Supernatants were eliminated and cell pellets were resuspended in ice-cold cytosolic extraction buffer (CEB, Pierce). After 10 min at 4°C, tubes were centrifuged at 500 × g for 5 min. Supernatants (corresponding to cytosolic fractions) were collected. Pellets were resuspended in membrane extraction buffer (MEB, Pierce), vortexed and left 10 min at 4°C. Tubes were then centrifuged at 3000 × g for 5 min. Supernatants (corresponding to membrane fractions) were collected and pellets were resuspended in nuclear extraction buffer (NEB, Pierce), giving the nuclear fraction. Fractions corresponding to cytosol, membrane and nuclei were boiled 5 min at 95°C in the presence of Laemmlli sample buffer. Fractions were then subjected to SDS-PAGE on 10% polyacrylamide gels and transferred onto nitrocellulose membranes. The membranes were saturated for 1 h at room temperature with saturation buffer (PBS containing 5% non-fat dried milk). After one wash with washing buffer (PBS supplemented with 0.1% Tween-20), membranes were incubated for 1 h with the appropriate primary antibody diluted in saturation buffer according to manufacturer's suggestions. After three washes with washing buffer, membranes were incubated for 1 h at room temperature with secondary antibody conjugated to alkaline phosphatase (from Jackson Immunoresearch) diluted in saturation buffer. Finally, membranes were washed six times and the immune complexes were detected using alkaline phosphatase substrate (NBT/BCIP from Pierce). The purity of each fractions and the absence of cross-contaminations were always assessed using antibodies directed against markers of each compartment (Hsp90, EGF-R and Histone H3, respectively).

### Measurement of the transport of glutamate

Cells originating from 75 cm^2 ^flasks were seeded onto 12-well CellBind™ plates at an initial density of 100.000 cells per cm^2^. The uptake, the secretion and the net transport of glutamate were measured on sub-confluent or confluent cells. Concentration of L-glutamate was measured using enzymatic quantification. The uptake of L-glutamate was measured as previously described [21,22]. Briefly, cells were washed with warmed Hank's balanced salt solution (HBSS; pH 7.4) supplemented with 10 mM D-glucose. After equilibration (15 min at 37°C in the CO_2 _incubator), medium was aspirated and 500 μL of HBBS + D-Glc containing 100 μM of L-glutamate was added. After 60 minutes at 37°C, media were collected and the residual extracellular glutamate concentrations measured using the enzymatic quantification kit (Amplex^® ^red glutamate assay from Invitrogen) [21,22]. Glutamate secretion was measured in DMEM medium containing L-cystine (200 μM) but no phenol red or glutamate. Cells were incubated for 6 h at 37°C in the CO_2 _incubator before extracellular glutamate concentration was enzymatically measured. Both uptake and secretion of L-glutamate were normalized using protein content of the wells determined by the Folin procedure [23]. Finally, the net transport of glutamate resulting from the activity of EAATs and X_c_^- ^was also measured. In that case, cells seeded onto 12-well CellBind™ plates were incubated at 37°C in the CO_2 _incubator with 500 μL of DMEM medium without phenol red but with L-cystine (200 μM) and L-glutamate (100 μM). After 6 h of incubation, media were collected and the concentrations of glutamate were measured as described above.

### Statistical analysis

t-Test was used to address the significant differences between mean values with significance set at *p *< 0.05 (GraphPad ^® ^Prism5 software).

## Results

### Localization of EAATs in human astrocytoma and primary astrocytes

**Figure 1:**
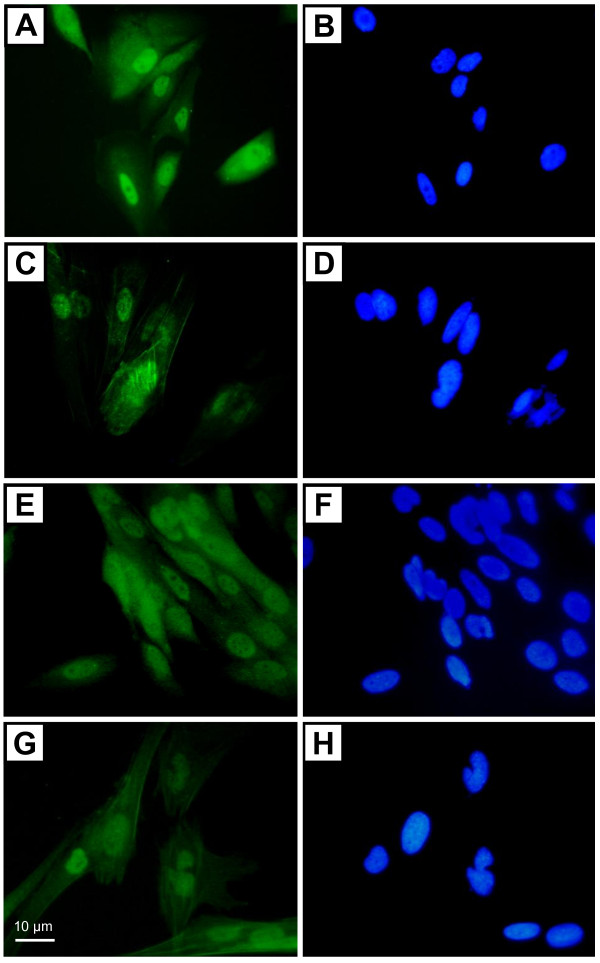
**Immunostaining of EAATs in sub-confluent human astrocytoma cells**. Sub-confluent STTG-1 cells were labelled for EAAT1/GLAST (**A**, **B **and **E**, **F**) or EAAT2/GLT-1 (**C**, **D **and **G**, **H**) using primary antibodies obtained from Santa Cruz (directed against N-terminal part of GLAST or GLT-1) (**A **and **C**) or from Chemicon (directed against the C- terminal part of GLAST or GLT-1) (**E **and **G**). Nuclei were stained with DAPI (**B**, **D**, **F**, **H**).

**Figure 2:**
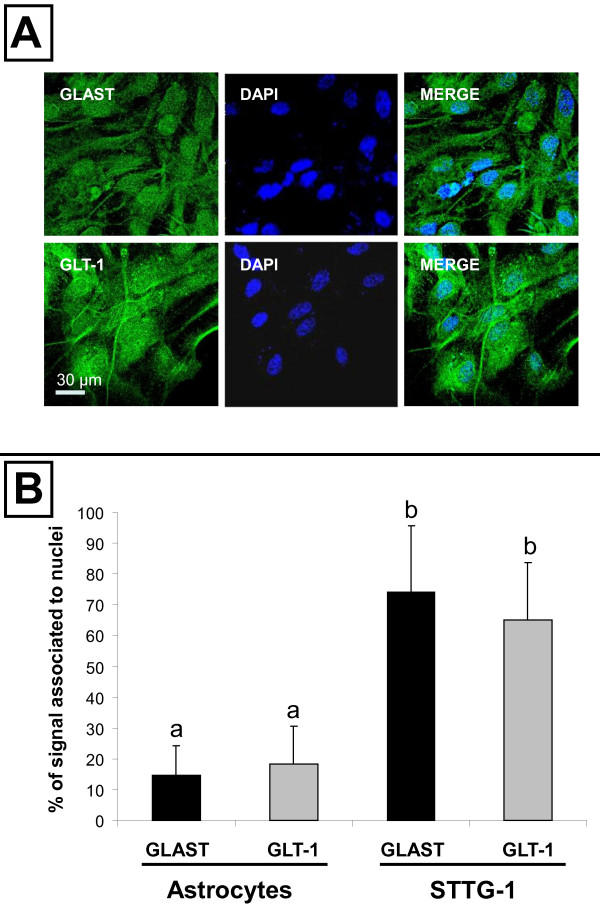
**Immunostaining of EAATs in sub-confluent primary astrocytes**. **A**- Primary astrocytes were labelled for EAAT1/GLAST or EAAT2/GLT-1. **B**- The percentage of the EAATs signal that co-localised with DAPI in normal astrocytes and STTG-1 cells was quantified using ImageJ as explained in Materials and Methods. Results obtained by quantification of at least 20 different cells were expressed as means ± standard deviation (SD). Bars without a common letter differ by at least *p *< 0.05.

STTG-1 cells were used to study EAATs localization in human astrocytomas as it was already demonstrated that this cell line perfectly mimics in vivo situation and is a valuable model of human astrocytomas [13]. Immunofluorescence (IF) microscopy, cell fractionation and transport studies confirmed that EAATs were associated with the nuclei in sub-confluent STTG-1 cells. Thus, IF results demonstrated that most of the EAATs signal co-localised with DAPI (Figure 1 and 2B). Changing the primary (directed against the N or the C-terminal part of EAATs) and secondary antibodies (directed against guinea pig or rabbit primary antibodies) did not significantly modify the co-localization of EAATs with DAPI (Figure 1). The immunostaining of EAATs in normal brain astrocytes furthermore confirmed that the aberrant localization of EAATs was restricted to malignant astrocytes (Figure 2). Finally, we labelled antigens specific of different cellular compartments: the cell membrane (i.e. EGF receptor), the cytosol (i.e. actin) and the nucleus (i.e. the transcription factor SP-1 and Histone 3) (data not shown). As expected, only histone 3 and SP-1 were mostly associated to the nuclei as demonstrated by the co-localization of histone 3/SP-1 and DAPI staining.

**Figure 3:**
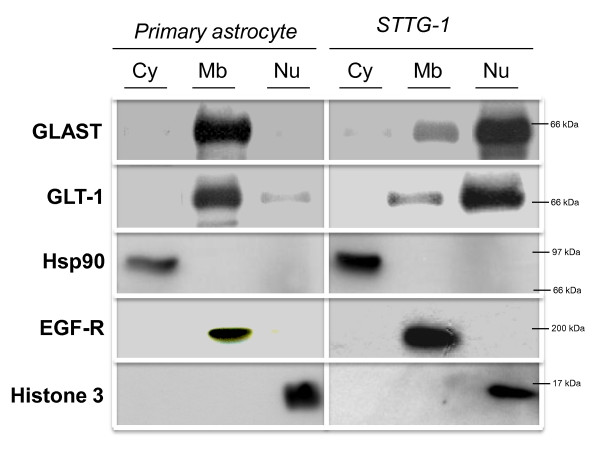
**Cell fractionation of primary and malignant astrocytes**. Sub-confluent primary astrocytes and STTG-1 cells were subjected to cell fractionation and western-blot analysis as explained in Materials and Methods. Nitrocellulose membranes were probed with antibodies directed against EAAT1/GLAST or EAAT2/GLT-1 or against antigens specific of each compartment (i.e. Hsp90, EGF-R and Histone 3 for the cytosolic, membrane and nuclear fractions, respectively). Cy = cytosolic fraction, Mb = membrane fraction, Nu = nuclear fraction.

Cell fractionation and western analysis confirmed the IF observations (Figure 3). Results demonstrated that EAATs were mainly associated to the membrane fraction and the nuclear fraction in primary astrocytes and STTG-1 cells, respectively.

Finally, EAATs mislocalization in human astrocytoma was demonstrated using transport studies (Table 2 and 3). Thus, the uptake of glutamate by sub-confluent STTG-1 cells was very low (at least 10 folds difference) compared to normal astrocytes (Table 2). Importantly, the use of a specific inhibitor of EAATs (i.e. L-trans-Pyrrolidine-2,4-dicarboxylic acid or PDC at 100 μM) confirmed the dependency of the uptake of glutamate to EAATs both in normal astrocytes and STTG-1 cells. The measurement of the secretion of L-glutamate showed that STTG-1 cells but not normal astrocytes were able to secrete L-glutamate through X_c_^- ^activity as demonstrated by the inhibition of the transport by sulfasalazine (250 μM) (Table 2). As a consequence of the defect of EAATs activity and of the increased activity of X_c_^-^, we found that STTG-1 cells were responsible for a net secretion of L-glutamate whereas normal astrocytes were responsible of a net absorption of glutamate (Table 3).

**Table 2:** Uptake and secretion of L-glutamate by human cancer cells and rat astrocytes.

	*Glutamate uptake*				*Glutamate secretion*			
	
	Sub-confluent cells		Confluent cells		Sub-confluent cells		Confluent cells	
	**control**	**+PDC**	**control**	**+PDC**	**control**	**+Sulfa**	**control**	**+Sulfa**

**AGS**	1.2 ± 2.7	0.7 ± 1.9	89 ± 15.2	***1.9 ± 3.3***	18 ± 4.7	***0.9 ± 2.3***	0.1 ± 0.9	1.2 ± 0.7

**Caco-2**	1.7 ± 1.9	0.8 ± 2.4	133 ± 19	***0.8 ± 1.1***	21 ± 1.8	***1.1 ± 0.7***	0.7 ± 1.3	1.4 ± 0.9

**HeLa**	6.1 ± 9.1	0.3 ± 2.2	92 ± 21	***2.1 ± 0.9***	24 ± 9.1	***1.0 ± 0.8***	0.9 ± 1.1	1.1 ± 0.8

**HOG**	1.7 ± 3.2	0.8 ± 0.9	86 ± 9.8	***1.3 ± 2.4***	19 ± 3.7	***0.7 ± 1.3***	0.4 ± 1.9	0.9 ± 1.2

**HT29**	0.8 ± 1.5	0.5 ± 2.1	121 ± 8.7	***1.7 ± 3.1***	27 ± 3.4	***0.8 ± 1.9***	0.3 ± 1.4	0.1 ± 1.9

**SHSY5Y**	18.4 ± 9.9	***0.7 ± 2.8***	84 ± 14	***0.9 ± 2.3***	14 ± 3.1	***1.1 ± 2.4***	0.1 ± 0.8	0.3 ± 2.1

**STTG-1**	1.6 ± 0.7	0.3 ± 1.3	2.1 ± 1.1	0.7 ± 0.9	34 ± 5.6	***0.2 ± 1.2***	42 ± 11	***1.1 ± 0.7***

**Astrocytes**	119 ± 12	***1.5 ± 8.4***	145 ± 27	***0.9 ± 2.7***	0.1 ± 0.3	0.0 ± 1.9	0.4 ± 1.2	0.1 ± 0.9

The uptake and the secretion of L-glutamate were measured as explained in Materials and Methods. Results were expressed as nmole of L-glutamate transported per mg of cellular proteins. Inhibitors used were PDC (100 μM) and sulfasalazine (250 μM). All results were obtained from three independent experiments and were expressed as means ± standard deviation (S.D). Values in ***bold ***are statistically different from the control values with *p *at least < 0.05.

**Table 3:** Net transport of L-glutamate by human cancer cells and rat astrocytes.

	*Sub-confluent cells*			*Confluent cells*		
	
	*control*	*+ PDC*	*+ Sulfa*	*control*	*+ PDC*	*+ Sulfa*
**AGS**	+ 32 (± 15)	+ 39 (± 21)	***- 9.1 (± 13)***	- 74 (± 6.1)	***+ 41 (± 18)***	- 81.2 (± 8.4)

**Caco-2**	+ 36 (± 26)	+ 44 (± 18)	***- 4.5 (± 9.2)***	- 69.9 (± 9.6)	***+ 42 (± 12)***	-72.6 (± 9.6)

**HeLa**	+ 27 (± 12)	+ 39 (± 9.8)	***- 7.8 (± 11)***	- 63.2 (± 5.8)	***+ 47 (± 18)***	- 69.1 (± 5.2)

**HOG**	+ 37 (± 9.6)	+ 41 (± 12)	***- 2.3 (± 3.4)***	- 68.4 (± 9.4)	***+ 52 (± 17)***	-71.7 (± 6.8)

**HT29**	+ 48 (± 14)	+ 51 (± 7.5)	***- 5.4 (± 9.1)***	- 64.1 (± 14)	***+ 49 (± 9.8)***	- 69.8 (± 5.9)

**SHSY5Y**	+ 15 (± 9.9)	***+ 34 (± 6.4)***	***- 13 (± 6.8)***	- 74 (± 1.8)	***+ 48 (± 13)***	- 79.2 (± 9.4)

**STTG-1**	+ 52 (± 24)	+ 62 (± 13)	***- 6.9 (± 3.4)***	+ 69 (± 12)	+ 74 (± 9.5)	***- 9.1 (± 7.9)***

**Astrocytes**	- 79 (± 2.1)	***+ 14 (± 10.9)***	- 82 (± 3.4)	- 91.7 (± 2.7)	***+ 21 (± 12)***	- 95.6 (± 9.1)

Net transport of L-glutamate was measured as explained in Materials and Methods. Results were expressed as variations of the extracellular concentration of L-glutamate after 6 h of incubation at 37°C. Initial concentration of L-glutamate in cell medium was 100 μM. Positive and negative variation values corresponded to net secretion and absorption of L-glutamate, respectively. Inhibitors used were PDC at 100 μM and sulfasalazine at 250 μM. All results were obtained from three independent experiments and were expressed as means of the variation (in μM) ± standard deviation (S.D). Values in ***bold ***are statistically different from the control values with *p *at least < 0.05.

### Localization of EAATs in non-astrocytoma human cancer cells

**Figure 4:**
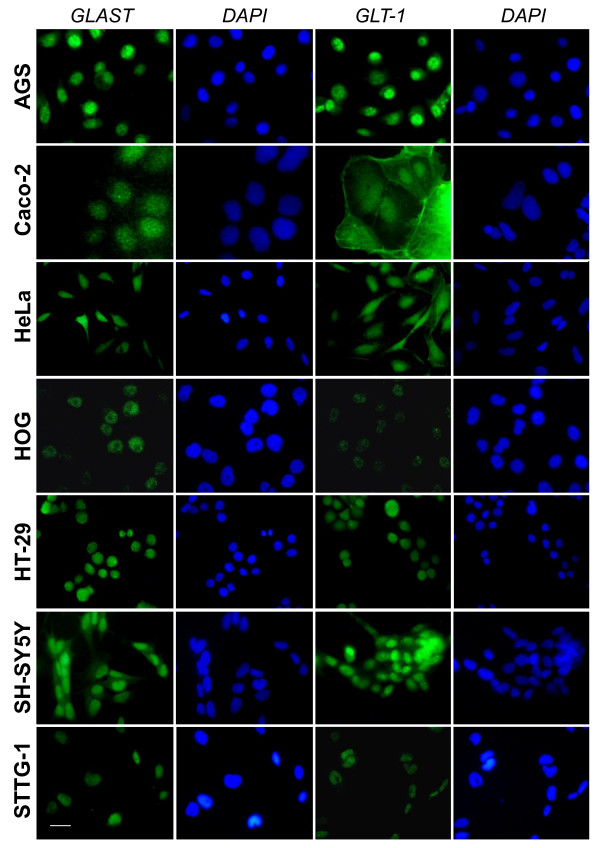
**Immunostaining of EAATs in sub-confluent human cancer cells**. Sub-confluent human cancer cells were labelled for EAAT1/GLAST or EAAT2/GLT-1. Nuclei were stained with DAPI.

**Figure 5:**
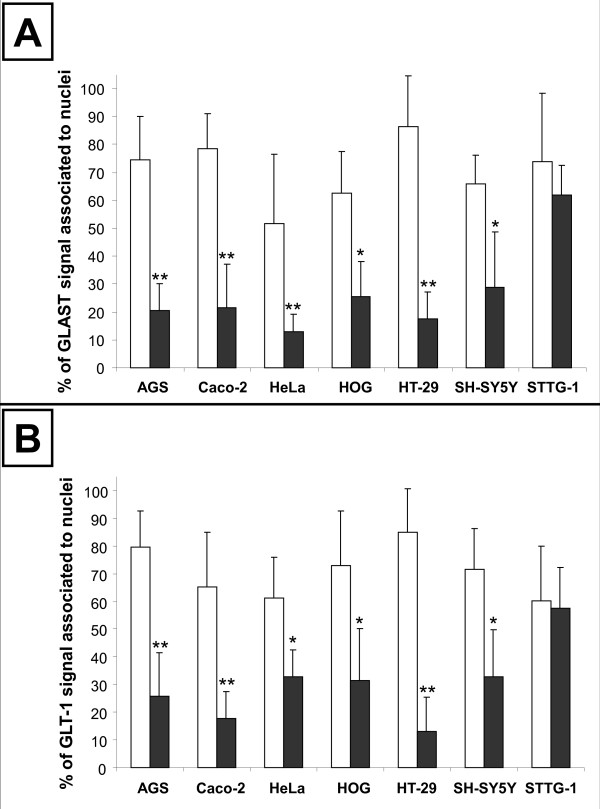
**Quantification of the nuclei-associated EAATs signal in sub-confluent and confluent human cancer cells**. Sub-confluent (white bars) and confluent (dark bars) human cancer cells were labelled for EAAT1/GLAST or EAAT2/GLT-1. The percentage of the GLAST (**A**) or GLT-1 (**B**) signal that co-localised with DAPI was quantified using ImageJ as explained in Materials and Methods. Results obtained by quantification of at least 20 different cells were expressed as means ± standard deviation (SD). Statistical difference between sub-confluent and confluent cells was evaluated using t-test with * *p *< 0.05 and ** *p *< 0.01.

The cellular localization of EAATs was then studied in various human non-astrocytoma cancer cells listed in Table 1. We first used sub-confluent cells at 1 or 2 days post seeding. Immunofluorescence microscopy showed that in all human cancer cells tested EAATs were mostly located in the nucleus (Figure 4 and 5). As for STTG-1 cells, changing the primary and secondary antibodies did not affect the labelling demonstrating that the observed mislocalization was not an artefact due to antibodies (data not shown).

**Figure 6:**
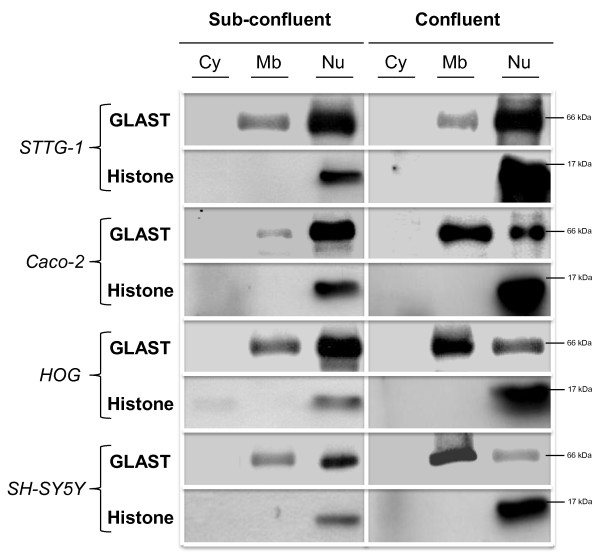
**Cell fractionation of sub-confluent and confluent human cancer cells**. Astrocytoma brain cancer (STTG-1), adenocarcinoma (Caco-2) and non-astrocytoma brain cancer (SH-SY5Y and HOG) cells were used. Sub-confluent and confluent human cancer cells were subjected to cell fractionation and western-blot analysis as explained in Materials and Methods. Nitrocellulose membranes were probed with antibodies directed against GLAST or Histone 3. Cy = cytosolic fraction, Mb = membrane fraction, Nu = nuclear fraction. Similar results were obtained with antibodies directed against GLT-1 (data not shown).

Cell fractionation (Figure 6) and transport studies (Tables 2 and 3) confirmed that EAATs were mostly mislocalized in all sub-confluent human cancer cells tested, all cancer cells, being unable to absorb L-glutamate and being responsible instead of a net secretion of it, due to the nuclear localization of EAATs.

### The cell confluence corrects the mislocalization of EAATs in all human cancer cells, except astrocytoma

**Figure 7:**
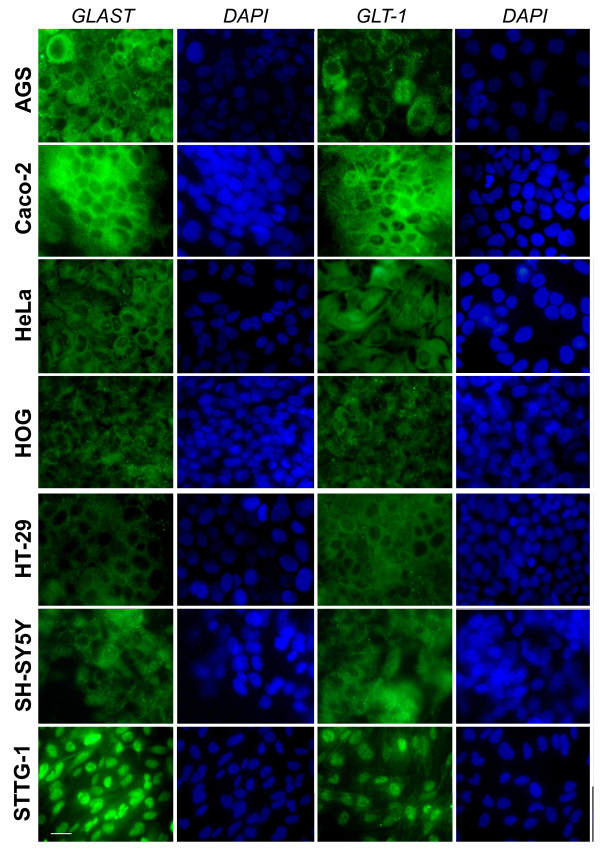
**Immunostaining of EAATs in confluent human cancer cells**. Confluent human cancer cells were labelled for EAAT1/GLAST or EAAT2/GLT-1. Nuclei were stained with DAPI.

Since previous publication has demonstrated that confluent Caco-2 cells absorb L-glutamate through EAATs activity [18], we decided to study the effect of the cellular confluence on the mislocalization of EAATs in human cancer cells. Cells were left for 7-10 days to obtain full confluence. Immunofluorescence microscopy showed that in all human cancer cells tested, except astrocytoma cells, cell contact caused the relocalization of EAATs out of the nuclei (Figure 5 and 7). Among the human cells tested, only Caco-2 undergoes cell differentiation after confluence, whereas all the other retain their undifferentiated status even after full confluence. Since EAATs relocalization was observed with all human cancer cells tested, except astrocytoma cells, it suggested that the cell differentiation was not involved in such process. To confirm that the relocalization of EAATs observed after 7 to 10 days was not linked to cell ages, we also seeded cells at high density in order to reach confluence faster, i.e. in 1-2 days. Similar relocalization of EAATs in all cancer cells tested except astrocytoma cells were obtained, suggesting that the phenomena was only dependent of cell confluence and cell-cell contacts (data not shown).

In order to confirm immunostaining results, the transport of L-glutamate by sub-confluent and fully confluent cells were compared (Tables 2 and 3). Results of uptake measurements showed that all human cell lines tested, except STTG-1, became able to absorb L-glutamate after cell confluence. Restored absorption was dependent of EAATs as demonstrated by the inhibitory effect of PDC. This observation confirmed the immunostaining results and demonstrated that in all human cancer cells tested, except astrocytoma, cell contact caused the functional relocalization of EAATs at the plasma membrane. As a consequence of the restoration of the EAATs activity, all confluent human cancer cells, except STTG-1, became responsible for a net absorption of L-glutamate (Table 3).

The relocalization of EAATs induced by cell contact was finally demonstrated using cell fractionation and western-blot analysis (Figure 6). The cell contact triggered the relocalization of part of the EAATs from the nuclear fraction to the membrane fraction in all the human cancer cells tested except astrocytoma.

## Discussion

Alterations of the handling of L-glutamate by astrocytomas are linked to major consequences on cancer growth, invasiveness and resistance to conventional treatments [4-7]. Indeed, whereas normal astrocytes are responsible for a net absorption of L-glutamate, malignant astrocytes secrete high levels of glutamate at the vicinity of the tumor. Such aberrant handling of glutamate by astrocytomas is related to an increase in the secretion of glutamate through the cystine/glutamate exchanger X_c_^- ^and a decrease in its absorption through the glutamate transporters EAATs. Pioneer studies have demonstrated that the compromised uptake of glutamate by astrocytoma cells is due to a nuclear mislocalization of the EAATs [13]. Thus, whereas EAATs are expressed at the plasma membrane of normal astrocytes, the transporters are associated to the nuclei in malignant astrocytes [13]. Due to the major role of extracellular glutamate on astrocytoma biology, strategies have been developed to tackle the cancer at this level. Thus, in vitro and in vivo experiments have demonstrated that blocking the activity of X_c_^- ^and/or the receptors of glutamate, could reduce the growth, the invasiveness and/or the resistance to conventional treatments of astrocytomas [7,15]. However, no strategies have been developed to correct the defect of glutamate uptake observed in human astrocytomas. One reason of this could be the fact that, at present, the mechanism responsible for the mislocalization of EAATs in malignant astrocytes is not identified.

Although EAATs are also expressed in non-astrocytoma cancers [18,19] and that alterations of the handling of L-glutamate have been observed in non-brain cancers [19], no one have looked at the cellular localisation of EAATs in non-astrocytoma cancers.

We found that EAATs are mislocalised in all sub-confluent human cancer cells originated from different tissues that we tested. Thus, EAATs are associated with the nuclei not only in human astrocytoma cells but also in human neuroblastoma, oligodendrocytoma and adenocarcinoma (from intestine, stomach or genital tract) cells. The mislocalization of EAATs in all sub-confluent human cells tested was first demonstrated by immunofluorescence microscopy and was furthermore confirmed by cell fractionation and by transport studies. This result demonstrates for the first time that the mislocalization of EAATs and the associated alteration of handling of glutamate are not restricted to astrocytoma cells and are observed in many other human malignant cells.

Importantly, we found that the cell confluence corrects the mislocalization of EAATs in all human cancer cells tested except astrocytoma cells. Thus, immunofluorescence microscopy observations demonstrated that cell confluence causes the relocalization of the EAATs signal out of the nuclei in adenocarcinoma, oligodendrocytoma and neuroblastoma, but not in astrocytoma cells. Cell fractionation and western-blot analysis confirmed immunofluorescence observations. Functional expression of EAATs at the plasma membrane was furthermore confirmed by transport studies that showed that, after cell confluence, all human cell lines tested became able to absorb L-glutamate whereas astrocytoma cells still secreted it.

Although additional studies are still required, our observations already suggest that modifications of the handling of L-glutamate by malignant cells could have implications in the biology (growth, invasiveness and resistance to treatment) of cancers other than astrocytomas as previously suggested by others [19]. Importantly, it was recently confirmed by silencing the expression of selected glutamate receptors that extracellular glutamate modulates the growth of non-astrocytoma cancer cells [24].

Although no EAATs mislocalization should be found in vivo in well developed/confluent non-astrocytoma human tumors, altered glutamate handling could be hypothetically found when non-astrocytoma cancer cells are not fully confluent, i.e. very early during tumor formation and/or when malignant cells leave the solid tumor as metastasis. Future studies using non-astrocytoma human malignant tissues at different stages of tumor growth should confirm our hypothesis.

The fact that EAATs are relocalized after cell confluence, at least in human non-astrocytoma cells, suggests that a cell-cell contact event triggers such phenomena. Future studies based on the comparison of cellular events caused by cell contact in human non-astrocytoma and astrocytoma cells should help characterising the membrane-associated molecules (protein(s) and/or lipid(s)) responsible for the correction. Interestingly, it has to be noted that mutations of the cadherin-related tumor suppressor homolog precursor also known as FAT tumor suppressor is frequently found in human astrocytomas [25]. Based on the cell surface expression of cadherins and their role in cell-cell contacts, such protein will be a good candidate.

## Conclusions

Taken together, our results demonstrated that the mislocalization of EAATs, initially observed with human astrocytomas, is not limited to this cancer type and happens in many others malignant cells, underlying a potential role of altered glutamate handling in those cancers, as described for astrocytomas. In addition, our results demonstrated that a cell-cell contact corrects the mislocalization of EAATs in all human cancer cells tested, except astrocytoma, suggesting that the cell contact-dependent signal allowing EAATs relocalization is absent in astrocytomas.. Identification and correction of this signal deficient in astrocytoma will potentially lead to the elaboration of new therapeutic strategies to correct EAATs defect and glutamate handling in astrocytoma depriving the tumor of the extracellular glutamate that is essential for its growth, invasion and resistance.

## Competing interests

The authors declare that they have no competing interests.

## Authors' contributions

KV, AB, CDS, NT, AA, SG and MM performed experiments. MM coordinated the study and wrote the manuscript. All authors read and approved the final manuscript.
